# Theoretical Stable Hydraulic Section based on the Principle of Least Action

**DOI:** 10.1038/s41598-019-44347-4

**Published:** 2019-05-28

**Authors:** Noriaki Ohara, Katsu Yamatani

**Affiliations:** 1 Department of Civil and Architectural Engineering, University of Wyoming, 1000 E. University Avenue, Laramie, WY 82071 USA; 2 Department of Urban Science, Meijo University, 4-102-9 Yataminami, Higashi, Nagoya, 461-8534 Japan

**Keywords:** Geomorphology, Sedimentology, Hydrology

## Abstract

Despite decades of effort, stable hydraulic geometry for an open channel water flow has hardly been established because of too many unknown variables for too few rational relationships. This article derives the most efficient channel cross section using calculus of variations for the given flow area at the minimum wetting perimeter length, which is equivalent to the principle of least action. Analysis indicates that water can most efficiently flow in a semi-ellipse section channel with minimum friction and erosion. Anisotropy in channel erodibility was found to be necessary in the natural stable channel characterization because gravitation force and channel bank consolidation cannot be ignored in earth surface material. This channel cross section, based on the principle of least action, may be regarded as the theoretical stable hydraulic section for erodible bed, which was comparable to the observed river cross-sections during high flow periods.

## Introduction

Stable river channel has been studied for almost a century and no consensus has been reached among the researchers. The U.S. Bureau of Reclamation^1,2^ identified the importance of the natural stable hydraulic section to minimize channel erosion and to stabilize manmade channels.

A standard characterization of the river channel section, known as regime equation, was established early in the1950s using power functions of discharge *Q*, as follows:

1 $$W\propto {Q}^{b}$$

2 $$D\propto {Q}^{{\epsilon }}$$

where *W* is the width of the river, and *D* is the depth of water at the center, and *b* and ${\epsilon }$ are the exponents. The work by Leopold, L. B., & Maddock^3^ popularized this formulation through analysis of the measured natural river geometry. However, the fundamental formulation may have originated much earlier^4^ and was presented as the regime theory for engineering practice by Blench^5^. This formulation has been accepted because the exponents were somewhat consistent (typically within 0.2–0.6) among the river measurements around the world^6^.

The channel cross section in the formulation has been assumed using an arbitrary shape, typically chosen from rectangle or trapezoid for convenience. Some textbooks^7,8^ described a cosine function for the stable hydraulic section, derived by Koechlin^9^ and Lane^2^. Pokhsraryan^10^ also proposed the trigonometric polynomial function for the stable hydraulic section for an erodible canal by using the force balance for a particle on the surface of the bed employing a few simplifications. However, the first order approximation, a cosine function, tends to be very shallow near the riverbanks even when it is applied to the wide river channel. The submerged angle of repose on the bed is an essential model parameter, which is hard to obtain during storm periods. Moreover, the lateral slope of the riverbed is often steeper than the angle of repose due to the riparian plant roots and the soil consolidation. Hirano and Aniya^11^ derived the cross-profile of glacial valleys as a catenary using variational calculus; however, the isoperimeter (constant perimeter) assumption was weakly justified. Nevertheless, as the catenary function can fit well with the glacial valley topography with some appropriate parameters, it might be possible to apply it to the river channel bed geometry. A few studies (e.g.)^12^ continued searching for a stable hydraulic section under a predetermined function type while there is no guarantee for the “best” hydraulic section for the erodible channel bed. Consequently, the geometry of the stable hydraulic section remains unanswered.

Essentially, there are too many unknowns (e.g. water depth, river width, channel slope, roughness, and river cross-sectional geometry) and too few equations (e.g. mass conservation, friction law, and sediment transport equation) even for a steady uniform flow. Therefore, additional relationships such as Maximum Flow Efficiency (MFE)^13^, Maximum Sediment Transport Capacity (MSTC)^14^, and Minimum Stream Power (MSP)^15^, have been proposed since the 1950s to solve the problem. Singh^6^ provided a good review on the developed theories and principles during the second half of the 20^th^ century in the area of hydraulic geometry, while none of them were truly conclusive for the channel design. The multitude of principles often lead to different conclusions and result in doubt about the existence of the universal steady hydraulic section.

Huang and Nanson^13^ showed that the several principles, including MFE, MSTC and MSP, can be unified into the principle of least action. This principle is sometimes referred to as the Maupertuis’s principle, which states that “Nature is thrifty in all its actions”, and has been used in modern physics to obtain the equation of motion for a system. The principle of least action in open channel hydraulics may be interpreted to mean that water must travel with least resistance and erosion. Therefore, this principle may be used to find stable channel geometry. In other words, the stable hydraulic section should be equivalent to the best hydraulic section that minimizes friction energy loss and channel erosion for a given flow area. This study explores theoretical stable hydraulic section based on the principle of least action using the variational calculus approach. The calculus of variations is a mathematical technique to find maxima and minima of functionals using differentiation of functions and functionals instead of variables. To date, there is no successful application of this technique to the stable hydraulic section analysis. This study will also discuss the anisotropic erodibility influence on the stable channel geometry.

## Derivation

**Figure 1:**
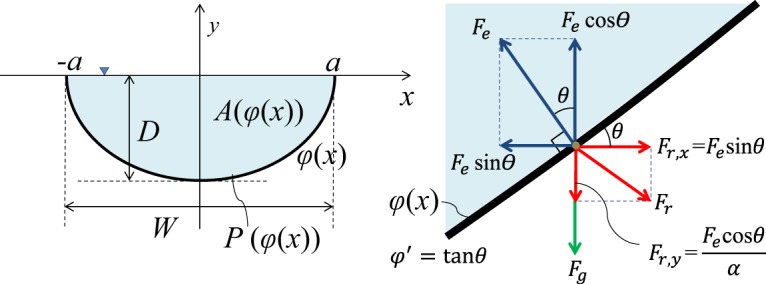
Schematic of the general channel cross section and forces acting on the channel bed.

The stable hydraulic section is defined as the section of an erodible channel in which no erosion will occur at a minimum water area for a given discharge^7^. This implies that the tractive force by the flow on the slope balances with the resistance force *F*_*r*_. Let *F*_*e*_ be the erosion force by flow traction and *F*_*r*_ the resistance force, as shown in Fig. 1. A general channel bed shape is considered as an arbitrary function $\phi (x)\in {C}^{2}$ while symmetricity is expected for a straight prismatic channel.

The required resistance for erosion toward the riverbank (horizontal directions) must be smaller than that toward the river bottom (vertical direction) because sediment at the channel bottom has to overcome gravity. In this study, the vertical erosion force on the flat riverbed requires *α* time more than the riverbed resistance force due to gravity. Hence, the resistance forces in the vertical and horizontal directions can be written as:

3 $${F}_{r,x}={F}_{e}\,\sin \,\theta ,\,{\rm{and}}$$

4 $${F}_{r,y}=\frac{{F}_{e}}{\alpha }\,\cos \,\theta .$$

Since the vertical erosion force balances with the summation of gravitational and vertical resistance forces, the parameter *α* can be written as:

5 $$\alpha =1+\frac{{F}_{g}}{{F}_{r,y}}.$$

Note that the parameter *α* should be greater than one when the gravity effect is not negligible. From Equations (3) and (4), the combined resistance force acting on the particle on the channel bed *φ* can be expressed as follows:

6 $${F}_{r}=\sqrt{{{F}_{r,x}}^{2}+{{F}_{r,y}}^{2}}=\sqrt{{({F}_{e}\sin \theta )}^{2}+{(\frac{{F}_{e}}{\alpha }\cos \theta )}^{2}}=\frac{{F}_{e}}{\alpha \sqrt{1+{\phi }^{\text{'}2}}}\sqrt{1+{(\alpha \phi ^{\prime} )}^{2}},$$

where the prime sign (′) denotes a derivative with respect to *x*. The total resistance force *F*_*r*,*total*_ against the river channel erosion can be obtained by integrating along the river channel perimeter.

7 $${F}_{r,total}={\int }_{-a}^{a}\,{F}_{r}\sqrt{1+{\phi ^{\prime} }^{2}}\,{\mathbb{d}}x=\frac{{F}_{e}}{\alpha }{\int }_{-a}^{a}\sqrt{1+{(\alpha \phi ^{\prime} )}^{2}}\,{\mathbb{d}}x=\frac{{F}_{e}}{\alpha }P[\phi (x)]$$

where *P* is the weighted wetting perimeter of the flow section that

8 $$P[\phi (x)]={\int }_{-a}^{a}\,\sqrt{1+{(\alpha \phi ^{\prime} (x))}^{2}}{\mathbb{d}}x.$$

This expression can also be interpreted that *α* is the weighting factor on the vertical line element $\phi ^{\prime} (x)$ to incorporate anisotropy in the channel erodibility. Thus, when the erosion process (erosion force and parameter *α*) is reasonably uniform within the river section at the equilibrium flow, the wetting perimeter represents channel erosion as well as flow resistance.

It is well known that the conveyance of a channel section increases with an increase in the hydraulic radius (A/P) or with a decrease in the wetted perimeter (P). As such, maximum conveyance for a given channel section area will occur with the smallest wetting perimeter when water surface friction is neglected. Equation (5) verifies that the stable hydraulic section is equivalent to the best hydraulic section for the erodible channel, as empirically found in the literature (e.g.)^7^.

The channel section area *A* for a general channel bed shape may be written as follows:

9 $$A[\phi (x)]={\int }_{-a}^{a}\phi (x){\mathbb{d}}x.$$

To find the stable hydraulic section $\phi (x)$ that minimizes $P[\phi (x)]$ based on the calculus of variation, the functional *F* is defined as

10 $$F[\phi (x)]=kA[\phi (x)]+P[\phi (x)]={\int }_{-a}^{a}[k\phi +\sqrt{1+{(\alpha \phi ^{\prime} )}^{2}}]{\mathbb{d}}x,$$

where *k* is a constant. Let

11 $$f(\phi (x),\phi ^{\prime} (x))=k\phi +\sqrt{1+{(\alpha \phi ^{\prime} )}^{2}}.$$

The Euler-Lagrange Equation (e.g.)^16^ can be formulated as:

$$\frac{\delta F[\phi ]}{\delta \phi }=\frac{\partial }{\partial \phi }f(\phi ,\phi ^{\prime} )-\frac{{\mathbb{d}}}{{\mathbb{d}}x}(\frac{\partial }{\partial \phi ^{\prime} }f(\phi ,\phi ^{\prime} ))=0$$

12 $$=\,k-\frac{{\mathbb{d}}}{{\mathbb{d}}x}(\frac{{\alpha }^{2}\phi ^{\prime} }{\sqrt{1+{(\alpha \phi ^{\prime} )}^{2}}})=0.$$

Assuming a horizontally symmetric, straight, and uniform open channel, the solution of this differential equation can be obtained, as follows:

13 $$\phi (x)=-\sqrt{{(\frac{1}{k})}^{2}-{(\frac{x}{\alpha })}^{2}}+d.$$

where *k* and *d* are constants. This solution indicates that the stable hydraulic section should be an ellipse for a straight channel in an erodible material. With a fixed flow section area, the hydraulic radius (*A*/*P*) becomes maximum when the ellipse channel is half full (semi-ellipse) or $\alpha =a/D$ (see the supplement to this article). As *k* = 1/*D*, and *d* = 0, we obtain,

14 $$\phi (x)=-\,D\sqrt{1-{(\frac{x}{a})}^{2}},\,{\rm{or}}\,{(\frac{x}{a})}^{2}+{(\frac{\phi (x)}{D})}^{2}=1.$$

This analysis shows the stable hydraulic section to be a semi-ellipse, which is commonly seen in nature. It is important that neither empirical flow-resistance relationship (e.g. Manning formula) nor sediment transport formulas (e.g. DuBoys formula) were used to derive the optimum cross section function.

## Results

**Figure 2:**
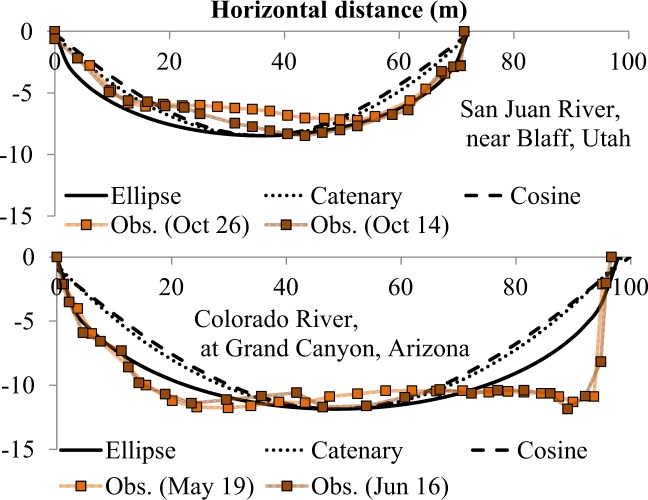
Comparisons of the theoretical (ellipse, catenary, cosine) and observed^3^ river cross-sections during the flood periods in 1941. The datum was set at the water surface during high flows.

Field observation of the stable hydraulic section is extremely difficult because the natural channel is never free from secondary flow due to meandering channel geometry and unsteady flow due to hydrological forcing. However, the stable hydraulic section may likely appear during the high-flow period when the riverbed is actively eroded; in fact, DuBoys formula expresses bed load transport rate as a quadratic function of hydraulic radius. Therefore, a “stable” hydraulic section may be achieved within a short period of time under a high flow condition. As such, we selected the river cross-sections measured by Leopold and Maddock^3^ on October 14 and 26, 1941, in the San Juan River near Bluff, Utah, and on May 19 and June 16, 1941, in the Colorado River at Grand Canyon, Arizona, because the dataset includes multiple surveys during the high flow periods, as shown in Fig. 2. The theoretical lines of ellipse (this study), catenary^11^, and cosine^10^ functions are compared against the flood time cross-sections of the San Juan (upper panel) and Colorado Rivers (lower panel). All theoretical lines were adjusted to meet the river width and depth at the high flow periods (Oct 14 and June 16, 1941 for the San Juan and Colorado Rivers, respectively). The statistics (root mean-square deviation, RMSD) are shown in Table 1. This analysis indicates that the ellipse cross-section seems more realistic than the other two functions (Fig. 2 and Table 1) to describe the river channel bathymetry at the high flow periods. It is interesting that the aspect ratios of flow sections in the two rivers were very close to each other during the high flow periods despite differences in river size and geographical location. Implications of the aspect ratio will be discussed further in the next section.

**Table 1:** Statistics for the comparison between theoretical and observed river cross sections.

	Date	Aspect ratio, ζ	Root-mean-square deviation (RMSD) (m)			
			Ellipse	Catenary	Cosine	N
San Juan R.	10/14/41	8.4	0.93	1.06	1.26	23
Colorado R.	6/16/41	8.2	2.05	3.36	3.52	26

The anisotropic erodibility parameter *α* simply appears as half of the aspect ratio ζ of the flow section, as can be expressed as,

15 $$\zeta =\frac{W}{D}=2\alpha .$$

Therefore, it can be directly estimated from the field data. When the Manning equation is used, the corresponding regime formulas may be computed as follows:

16 $$D={C}_{M}{Q}^{3/8},\,{\rm{and}}$$

17 $$W=2\alpha {C}_{M}{Q}^{3/8},$$

where

18 $${C}_{M}={[\frac{2n}{\pi \alpha }{(\frac{4K}{\pi })}^{2/3}{S}^{-1/2}]}^{3/8},\,{\rm{and}}$$

19 $$K=1+\frac{1}{2}(\mathrm{ln}\,4\alpha -\frac{1}{1\cdot 2})\frac{1}{{\alpha }^{2}}+\cdots .$$

The Cayley formula was selected for the wetting perimeter computation because of the large aspect ratio of the natural river channel^17,18^. Note that *K* is not a function of depth *D*. As such, when the Cayley formula is valid, the hydraulic radius is just proportional to the depth at the center.

According to the Equations (16) and (17), the exponents were found to be $b={\epsilon }=3/8$ for the stable channel in an equilibrium state when the Manning formula is valid. This is consistent with the numbers shown in Table 2.

**Table 2:** Estimated parameters for the regime formulas from the selected studies.

Aspect ratio, ζ	b	${\boldsymbol{\epsilon }}$	Investigators	Note
8.2, 8.4	0.26	0.40	^3^	Field, 20 river stations in the Great Plains, US
—	0.5	0.4	^3^	Field, over 20 rivers basins in the US
22.86	0.50	0.34	^22^	Field, Missouri River basin (252 gaging stations)
—	0.38	0.46	^20^	Field, Gwydir R., Namoi R., Barwon R.
32.7–61.5	0.40	0.38	^23^	Field, Sacramento R., Alberta R., Joganji R.
	0.500	0.333	^4, 5^	Theory, unit flow, regime theory, sectional equation
2.5	0.440	0.440	^13^	Theory, trapezoid, Lacey formula
2.5–30	0.478	0.289	^13^	Theory, trapezoid, Lacey formula
flexible	0.375	0.375	This study	Theory, ellipse, Manning formula

The field observations in Table 2 indicate that the regime theory holds for many different rivers all over the world despite tremendous variability in the aspect ratio and uncertainty in the field data. It is, in fact, theoretically obvious that the regime relationships were independent from the anisotropy of the channel erodibility.

A few studies (e.g.)^19,20^ have discussed some non-linear relationship between water depth and river width, typically in a power function which results in the inequality of the exponents *b* and ${\epsilon }$. The aspect ratio is arguably driven by the riverbed material, sediment composites, and geological history rather than the fluid mechanics. For example, the governing equation for the fluid mechanics, the Naiver-Stokes Equation, only weakly associates with asymmetry for incompressible fluid. Nanson and Huang^21^ characterized the aspect ratio of cross-section by excess bed shear which is a function of site-specific attributes such as slope, bed roughness, etc. at an equilibrium state. When the aspect ratio can be expressed as a site-specific power function of flow rate, the inequality of the exponents, *b* and ${\epsilon }$, is likely site-specific.

This theory also suggests that the channel cross section may approach a semi-circle as erosion force increases during the high flow period. According to Equation (5), the anisotropic erodibility parameter *α* becomes closer to one as the erosion force magnitude increases compared to the gravitational force. This may provide some explanation of round cross-section by debris flow with rapid erosion and U shape valley by glacial plastic flow when the effect of gravity in erosion process is relatively small. Again, this property makes it extremely difficult to find an elliptic cross-section in a natural river because the flow rate varies drastically. However, the elliptic cross-section may be a more reasonable assumption for dam breach analysis than rectangular, triangular, or trapezoidal cross-sections that have been commonly used.

## Conclusion

Here we present the simple fact that the stable hydraulic section is a semi-ellipse based on the principle of least action. We looked for the optimum flow section shape minimizing the wetting perimeter (water and earth contact surface) so that the water can flow with minimum flow friction and sediment mass transport. The stable channel cross section was derived by directly solving the Euler-Lagrange Equation, the main principle of variational calculus. The real river channels typically have very large aspect ratio with significant variability (aspect ratio of 5–100). To incorporate the anisotropy in the riverbank erodibility, the weighted wetting perimeter was introduced. The detailed derivation of the formula is detailed in the supplement. The mathematical analysis resulted in a semi-ellipse section minimizing the channel friction and erosion in the straight erodible bed, and the aspect ratio of the flow section is a direct indicator of anisotropy in the riverbank erodibility. This study may be the first successful application of the variational calculus for theoretically-derived stable hydraulic section.

The derived semi-ellipse flow section was compared against the field data, which were observed during the high flow periods although it is impossible to conclude this analysis due to unsteadiness of river flow and secondary flow caused by meandering. The new theory seemed more effective in describing the channel cross-sectional bathymetry especially near the banks than the existing trigonometric and catenary functions, which have different origins. This theory was also consistent with the regime theory that has been mainly used for a stable channel design. Moreover, this new theory can effectively characterize site-specific soil and regolith properties (e.g. anisotropic erodibility α) from field data as well as design a stable channel in practice. It may be noted that the elliptic stable hydraulic section was derived without using any empirical and experimental relationships, such as the Manning and DuBoys formulas. Consequently, this theory holds broad applicability to any open channel flow of various liquids on erodible material.

## Supplementary information

Theoretical Stable Hydraulic Section based on the Principle of Least Action
