# Supplementary material for: Theoretical Stable Hydraulic Section based on the Principle of Least Action
Source: Sci Rep. 2019 May 28;9:7957. doi: 10.1038/s41598-019-44347-4 (PMC6538677; doi:10.1038/s41598-019-44347-4)
Supplement: Supplementary file 1 — Theoretical Stable Hydraulic Section based on the Principle of Least Action [file 41598_2019_44347_MOESM1_ESM.docx]

**Theoretical Stable Hydraulic Section based on the Principle of Least Action**

Noriaki Ohara^1*^ and Katsu Yamatani^2^

^1^Department of Civil and Architectural Engineering, University of Wyoming, 1000 E. University Avenue, Laramie, WY 82071, USA. Tel: +1(307)343-2670. E-mail: nohara1@uwyo.edu

^2^Department of Urban Science, Meijo University, 4-102-9 Yataminami, Higashi, Nagoya 461-8534, Japan

This supplemental document provides the complete derivation of the general stable hydraulic section for readers interested in the detailed mathematical derivation. The polar coordinate system is chosen in this document for convenience.

# Weighted Wetting Perimeter using β-norm

The channel section may be expressed as an arbitrary function $\varphi\left( r \right)\in C^{2}$. When the function $\varphi\left( r \right)$ is assumed to be symmetric around the y axis (center of flow section), it is a monotonic function in $0<r<a$.

Figure S-1 - Schematic of the general channel cross section

As $\varphi$ is a continuous even function around *r* =0, we have

${\lim_{r\to0} \varphi}^{'}\left( r \right)=0$. (S-1)

Let *F_e_* be the erosion force and *F_r_* the resistance force, as shown in Figure S-1. It is assumed that a particle on the flat riverbed requires α time smaller resistance force than the erosion force due to gravity. On the other hand, the horizontal resistance force must be equal to the horizontal erosion force. Therefore, the resistance forces in the vertical and horizontal directions can be written as

$F_{r,x}=F_{e}\sin\theta$ (S-2)

$F_{r,y}=\frac{F_{e}}{\alpha}\cos\theta$ (S-3)

To evaluate the combined resistance force acting on the particle, the β-norm is introduced for the generalized geometry, as follows:

$\left\| F_{r} \right\|_{\beta}=\left[ {F_{r,x}}^{\beta}+{F_{r,y}}^{\beta} \right]^{\frac{1}{\beta}}$

$=\left[ \left( F_{e}\sin\theta\right)^{\beta}+\left( \frac{F_{e}}{\alpha}\cos\theta\right)^{\beta} \right]^{\frac{1}{\beta}}$

$=\left[ \left( \frac{F_{e}\varphi^{'}}{\sqrt{1+{\varphi^{'}}^{2}}} \right)^{\beta}+\left( \frac{F_{e}}{\alpha\sqrt{1+{\varphi^{'}}^{2}}} \right)^{\beta} \right]^{\frac{1}{\beta}}$

$=\frac{F_{e}}{\alpha\sqrt{1+{\varphi'}^{2}}}\left[ \left( \alpha\varphi' \right)^{\beta}+1 \right]^{\frac{1}{\beta}}$ (S-4)

where the prime sign ($'$) denotes a derivative with respect to $r$. The parameter *β* for combining resistance force components, which is a constant within $1\leq\beta\leq2$, provides one additional freedom in the possible theoretical curve. Particularly, when $\beta=2$, the resistance force is evaluated in the Euclidean space as presented in the main article. The resistance force may be integrated along the river channel bed (*Γ*).

$\int_{\Gamma} \left\| F_{r} \right\|_{\beta}\mathbb{d}\Gamma=\int_{0}^{a} \left\| F_{r} \right\|_{\beta}\sqrt{1+{\varphi^{'}}^{2}}\mathbb{d}r$

$=\int_{0}^{a} \frac{F_{e}}{\alpha\sqrt{1+{\varphi'}^{2}}}\left[ \left( \alpha\varphi' \right)^{\beta}+1 \right]^{\frac{1}{\beta}}\sqrt{1+{\varphi^{'}}^{2}}\mathbb{d}r$

$=\frac{F_{e}}{\alpha}\int_{0}^{a} \left[ \left( \alpha\varphi' \right)^{\beta}+1 \right]^{\frac{1}{\beta}}\mathbb{d}r$

$=\frac{F_{e}}{\alpha}P$ (S-5)

where $P$ is the weighted wetting perimeter of the flow section that

$P\left[ \varphi\right]=\int_{0}^{a} \left[ \left( \alpha\varphi' \right)^{\beta}+1 \right]^{\frac{1}{\beta}}\mathbb{d}r$. (S-6)

Equation (S-5) indicates the proportionality between the resistance force and the wetting perimeter length. As such, minimization of the wetting perimeter at a steady state is equivalent to the principle of least action. The corresponding flow section area in the coordinate system can be written as,

$A\left[ \varphi\right]=\int_{0}^{a} \varphi\mathbb{d}r$. (S-7)

# Variational Calculus

To find the optimum flow section$\varphi$ that has minimum wetting perimeter $P\left[ \varphi\right]$ with a given flow section area $A\left[ \varphi\right]$, the calculus of variation is used (e.g. Courant and Hilbert, 1954; Gelfand and Fomin, 1963). The functional *F* is redefined as

$F\left[ \varphi\right]=kA\left[ \varphi\right]+P\left[ \varphi\right]=\int_{0}^{a} \left[ k\varphi+\left\{ {1+\left( \alpha\varphi' \right)}^{\beta} \right\}^{1/\beta} \right]\mathbb{d}r$, (S-8)

where *k* is an arbitrary constant. Let

$f\left( \varphi,\varphi' \right)=k\varphi+\left\{ {1+\left( \alpha\varphi' \right)}^{\beta} \right\}^{1/\beta}$. (S-9)

To find the function $\varphi$ that minimizes the functional $F\left[ \varphi\right]$, the Euler-Lagrange Equation can be used.

$\frac{\delta F\left[ \varphi\right]}{\delta\varphi}=\frac{\partial}{\partial\varphi}f\left( \varphi,\varphi' \right)-\frac{\mathbb{d}}{\mathbb{d}r}\left( \frac{\partial}{\partial\varphi^{'}}f\left( \varphi,\varphi^{'} \right) \right)=0$

$=k-\frac{\mathbb{d}}{\mathbb{d}r}\left( \frac{\alpha\left( \alpha\varphi' \right)^{\beta-1}}{\left\{ {1+\left( \alpha\varphi' \right)}^{\beta} \right\}^{1/\beta}} \right)=0$ . (S-10)

This differential equation can be solved as

$\frac{\alpha\left( \alpha\varphi' \right)^{\beta-1}}{\left\{ {1+\left( \alpha\varphi' \right)}^{\beta} \right\}^{1/\beta}}=kr+c$. (S-11)

From Equation (S-1), it is obvious that$c=0$. Then, this equation can be rearranged as,

$\varphi^{'}=\frac{\left( kr \right)^{\frac{1}{\beta-1}}}{{\alpha\left\{ \alpha^{\frac{\beta}{\beta-1}}-\left( kr \right)^{\frac{\beta}{\beta-1}} \right\}}^{1/\beta}}$. (S-12)

Considering the horizontal symmetricity (Equation S-1), this differential equation can be solved as:

$\varphi=-\left\{ \left( \frac{1}{k} \right)^{\frac{\beta}{\beta-1}}-\left( \frac{r}{\alpha} \right)^{\frac{\beta}{\beta-1}} \right\}^{\frac{\beta-1}{\beta}}+d$, or

$\left( \frac{r}{\alpha} \right)^{\frac{\beta}{\beta-1}}+\left( d-\varphi\right)^{\frac{\beta}{\beta-1}}=\left( \frac{1}{k} \right)^{\frac{\beta}{\beta-1}}$ (S-13)

which is a super-ellipse (Lamé curve), where *k* and *d* are constants. The solution was visualized in Figure S-2.

Figure S-2 – Super-ellipse curves as generalized stable hydraulic section (*β=*1.2, 1.5, 1.8, and 2.0)

Note that the non-Euclidean geometry ($1<\beta<2$) may represent the profile affected by three-dimensional flow in a river channel possibly due to meandering. Hence, when a straight and uniform open channel is considered, this equation can be reduced to the Euclidean case ($\beta=2$) as follows:

$\varphi\left( x \right)=-\sqrt{\left( \frac{1}{k} \right)^{2}-\left( \frac{r}{\alpha} \right)^{2}}+d$, (S-14)

which is an ellipse.

# Optimum Water Depth in a Ellipse Channel at an equilibrium state

If it is assumed that the isotropic erosion parameter $\alpha$ stays constant at the steady (equilibrium) state, the most effective water depth in an ellipse channel can be determined. Equations (S-13) can be rewritten using the angle $\theta$ re-defined in Figure S-3.

$\varphi=-R\left\{ 1-\left( \frac{r}{\alpha R} \right)^{u} \right\}^{\frac{1}{u}}+R\cos\theta$, or

$\left( \frac{r}{\alpha R} \right)^{u}+\left( \frac{R\cos\theta-\varphi}{R} \right)^{u}=1$ (S-15)

where

$R=\frac{1}{k}$, and $u=\frac{\beta}{\beta-1}$. (S-16)

So,

$R\cos\theta=d$. (S-17)


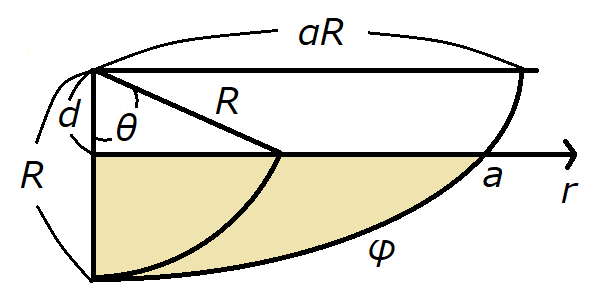


Figure S-3 – Definitions of the variables

Based on this expression, the flow sectional area A and the wetting perimeter P of the super-ellipse channel can be expressed as

$\left| A \right|=\alpha R^{2}\int_{0}^{N\left( \theta\right)} \left\{ \left( 1-t^{u} \right)^{\frac{1}{u}}-\cos\theta\right\}\mathbb{d}t$ (S-18)

$P=\alpha R\int_{0}^{N\left( \theta\right)} \left( 1-t^{u} \right)^{\frac{1}{u}-1}\mathbb{d}t$ (S-19)

where

$t=\frac{r}{\alpha R}$ , and

$N\left( \theta\right)=\frac{a}{\alpha R}=\left\{ 1-\left( \cos\theta\right)^{u} \right\}^{\frac{1}{u}}$ . (S-20)

Eliminating *R* from Equations (S-18) and (S-19) yields,

$P=\frac{\sqrt{\alpha\left| A \right|}\int_{0}^{N\left( \theta\right)} \left( 1-t^{u} \right)^{\frac{1}{u}-1}\mathbb{d}t}{\sqrt{\int_{0}^{N\left( \theta\right)} \left\{ \left( 1-t^{u} \right)^{\frac{1}{u}}-\cos\theta\right\}\mathbb{d}t}}$ . (S-21)

At $\beta=2$, this equation can be reduced to

$P=\frac{\theta\sqrt{2\alpha\left| A \right|}}{\sqrt{\theta-\cos\theta\sin\theta}}$. (S-22)

Clearly, the wetting perimeter *P* reaches the minimum at $\theta=\pi/2$ by setting the flow section area constant. Thus, the theoretical stable hydraulic section is a semi-ellipse (half ellipse) for a straight channel in an erodible material. In this case, we have $\alpha=\frac{a}{D}, k=1/D$, and $d=0$ . Accordingly, Equation (S-14) can be reduced to

$\varphi\left( x \right)=-D\sqrt{1-\left( \frac{r}{a} \right)^{2}}$, or $\left( \frac{r}{a} \right)^{2}+\left( \frac{\varphi}{D} \right)^{2}=1$. (S-23)


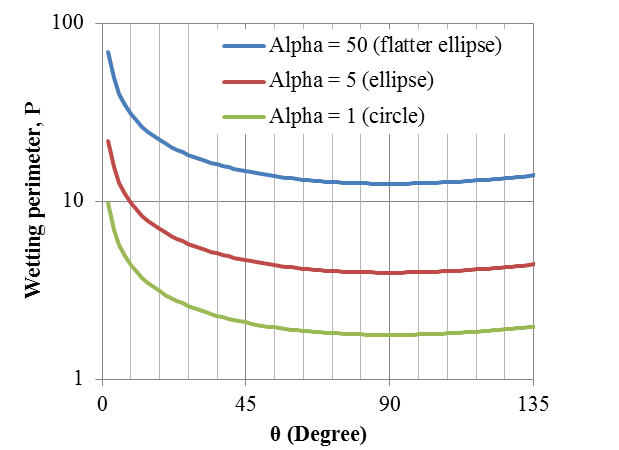


# References

Courant, R., & Hilbert, D. Methods of mathematical physics, vol. I. *Physics Today*, *7*(5), 17-17 (1954).

Gelfand, I. M., & Fomin, S. V. Calculus of variations. Revised English edition translated and edited by Richard A. Silverman. *Prentice Hall, Englewood Cli s, NJ*, *7*, 10-11 (1963).
